# Evaluation of the clinical signs of anterior cruciate ligament and meniscal injuries

**DOI:** 10.4103/0019-5413.55466

**Published:** 2009

**Authors:** Dhavalakumar K Jain, Rajkumar Amaravati, Gaurav Sharma

**Affiliations:** Department of Orthopaedics, St. John's National Academy of Health Sciences, Bangalore, India

**Keywords:** Anterior cruciate ligament, meniscus, tests for anterior cruciate ligament and meniscus

## Abstract

**Background:**

The diagnostic accuracy of anterior drawer (AD) sign, Lachman test and the pivot shift test for anterior cruciate ligament injury and McMurray test for medial and lateral meniscus is varied with sensitivity and specificity ranging from 2 to 100%. Generally, it is accepted that the pivot shift test is the most specific test to diagnose anterior cruciate ligament (ACL) tears and that the Lachman test is more sensitive than AD sign. This study was undertaken to calculate the sensitivity, specificity, positive predictive value, negative predictive value, and efficiency for the above-mentioned diagnostic tests.

**Materials and Methods:**

Twenty-eight male patients with clinical ACL injury were examined in the outpatient department and under anaesthesia, the findings were compared with arthroscopy.

**Result:**

The sensitivity and specificity for the Lachman test, AD sign and pivot shift test performed in the outpatient setting are 78.6 and 100%, 89.3 and 100%, and 75 and 100%, respectively. The sensitivity and specificity for the Lachman test, AD sign, and pivot shift test performed under anesthesia are 92.9 and 100%, 92.9 and 100%, and 100 and 100%, respectively. The sensitivity and specificity of the McMurray test for medial and lateral meniscus were 35.7 and 85.7% and 22.2 and 100%, respectively.

**Conclusion:**

The Lachman test, AD sign and pivot shift test are highly specific tests to diagnose ACL laxity in a non-acute setting; pivot shift test under anesthesia is the most sensitive and specific test for diagnosing ACL laxity in a non-acute setting and the McMurray test is not a sensitive test to diagnose meniscal injury in the presence of ACL injury.

## INTRODUCTION

The purpose of clinical examination is to make a correct diagnosis. In addition to taking a clinical history and physical examination, prompt assessment of the extent of knee damage may require further investigation.^1^ Several clinical tests have been described to clinically diagnose meniscal and anterior cruciate ligament (ACL) injuries. Using these tests, the ability to diagnose meniscal and ACL injuries reveals much disparity in accuracy.^2^,^3^ A thorough physical examination is the first step in evaluating a patient with knee pain.^3^ The McMurray test is the most common clinical test used to diagnose meniscal tears. Anterior drawer (AD) sign, Lachman test, and pivot shift test are commonly used to diagnose ACL injury. There is no uniformity in the results published about the accuracy of these tests. Generally, it is accepted that the pivot shift test is the most specific test to diagnose ACL tears and that the Lachman test is more sensitive than AD sign. Various authors have compared clinical tests with magnetic resonance imaging (MRI) and/or arthroscopy and the non-uniform nature in the accuracy is evident. The sensitivity and specificity of the tests used to diagnose ACL and meniscus injury range from 2 to 100%.^1^–^8^

We aim to compare the diagnostic accuracy of AD sign, Lachman test, and the pivot shift test performed in the outpatient setting and under anesthesia with arthroscopy findings. This study was undertaken to calculate the sensitivity, specificity, positive predictive value, negative predictive value, and efficiency for the above-mentioned diagnostic tests. In this study, we also aim to compare the diagnostic accuracy of the McMurray test for medial and lateral meniscus performed in an outpatient setting with arthroscopy findings.

## MATERIALS AND METHODS

This study is a retrospective study. The ethical clearance to conduct the study was obtained from the institutional review board.

Twenty-eight consecutive male patients with ACL injury of the knee with or without other associated ligament injuries of the knee were included in the study. The mode of injury was sports related in 14 (50%), road traffic accident in eight (28.5%), and others in six (21.5%). Presentation was instability alone in eight (28.5%), pain alone in one (3.6%), instability and pain in 15 (53.6%), and instability, pain and swelling in four (14.2%). The diagnosis was made from a combination of history and clinical examination. Skeletally immature patients with ACL injury were excluded from the study. All the subjects in our study had non-acute ACL injuries where more than 3 weeks had elapsed from the time of injury. All the patients in the study subsequently underwent ACL reconstruction using bone patellar tendon bone graft as they did not respond to physiotherapy regime. The patients included in the study were examined pre-operatively in the outpatient department (OPD) and the ligament laxity was re-examined on the operating table under anesthesia. These tests were performed by the same individual in the OPD and the operating theatre, who was not the operating surgeon. MRI of the knee was performed in patients who could afford the investigation. The indications for arthroscopy in our series were positive ACL laxity, positive McMurray test, giving way symptom, persisting pain, and persisting swelling. The above criteria were utilized to select patients for arthroscopy. The findings were entered in a standard proforma and the Lachman test, AD sign, pivot shift test, and McMurray tests were graded as positive or negative and retrieved at the time of the study.

### Statistical methods

Sensitivity, specificity, and related statistics were calculated in comparison of various procedures with various gold standards. Whenever all rows and columns were equal in number, the kappa of agreement was also computed. The data were analyzed for sensitivity, specificity, positive predictive value and negative predictive value.

#### Sensitivity

The ability of the test to identify the presence of disease. This is calculated by the formula, Sensitivity = True positives/(True positives + False negatives)

#### Specificity

The ability of the test to identify the absence of disease. This is calculated by the formula, Specificity = True negatives/(True negatives + False positives)

#### Positive predictive value

Proportion of cases that actually have the disease of the total number of cases predicted by the test to have the disease. This is calculated by the formula, Positive predictive value = True positives/(True positives + False positives)

#### Negative predictive value

Proportion of cases that actually are disease free of the total number of cases predicted by the test to be disease free. This is calculated by the formula, Negative predictive value = True negatives/(True negatives + False negatives)

#### Efficiency

Ability of the test to correctly identify presence and absence of disease, i.e., proportion of cases correctly classified.

## RESULTS

This study included 28 male patients with ACL injury, the mean age being 26.2 years (range 17–35 years). The right knee was affected in 17 (60.7%) patients and the left knee was affected in 11 (39.3%). Mode of injury was sports related in 14 (50%), road traffic accident in eight (28.5%), and others in six (21.5%).

The Lachman test carried out in the outpatient setting correctly identified ACL injury in 22 of the 28 patients and in six patients, it failed to detect ACL injury. There were no false positives or true negatives.

AD test carried out in the outpatient setting correctly identified ACL injury in 25 of the 28 patients and in three patients it failed to detect ACL injury. There were no false positives or true negatives.

Pivot shift test carried out in the outpatient setting correctly identified ACL injury in 21 of the 28 patients and in seven patients it failed to detect ACL injury. There were no false positives or true negatives.

The Lachman test carried out under anesthesia correctly identified ACL injury in 26 of the 28 patients and in two patients it failed to detect ACL injury. There were no false positives or true negatives.

AD sign under anesthesia correctly identified ACL injury in 26 of the 28 patients and in two patients it failed to detect ACL injury. There were no false positives or true negatives.

Pivot shift test performed under anesthesia correctly identified ACL injury in 28 patients. There were no false negatives, false positives or true negatives.

In our study, 10 (36%) patients had only medial meniscal tear, five (18%) patients had only lateral meniscal tear, and four (14%) patients had both medial and lateral meniscal tear.

McMurray test for medial meniscus performed in the outpatient setting correctly identified medial meniscus tear in five of the 14 patients. The test was false negative in nine patients, false positive in two patients, and true negative in 12 patients.

McMurray test for lateral meniscus performed in the outpatient setting correctly identified lateral meniscus tear in two of the nine patients. The test was false negative in seven patients, false positive in none, and true negative in 19 patients.

Table 1 gives the sensitivity, specificity, positive predictive value, negative predictive value, and efficiency of the above tests used for knee examination.

**Table 1:** Sensitivity, specificity, positive predictive value, negative predictive value, efficiency, positive likelihood ratio and negative likelihood ratio

	Sensitivity	Specificity	Positive predictive value	Negative predictive value	Efficiency	Positive likelihood ratio	Negative likelihood ratio
Lach OPD	78.6	100	100	0	78.6	∞*	0.21
Lach EUA	92.9	100	100	0	92.9	∞	0.07
AD OPD	89.3	100	100	0	89.3	∞	0.11
AD EUA	92.9	100	100	0	92.9	∞	0.07
Pivot shift OPD	75	100	100	0	75	∞	0.25
Pivot shift EUA	100	100	100	100	100	∞	0
McMurray Med Men	35.7	85.7	71.4	57.1	60.7	2.56	0.74
McMurray Lat Men	22.2	100	100	73.1	75	∞	0.78

* Infinity, Lach = Lachman test, OPD = Out patient department, EUA = Evaluation under anaesthesia, AD = Anterior drawer sign, Med = Medial, lat = Lateral, Men = Meniscus

## DISCUSSION

Fischer *et al*.^4^ made a multicenter analysis of 1014 patients. MRI of the knee was performed and the findings were subsequently confirmed arthroscopically. The accuracy of the diagnosis from the imaging was 89% for the medial meniscus, 88% for the lateral meniscus, 93% for the ACL, and 99% for the posterior cruciate ligament. Lee *et al*.^5^ reviewed 79 MR studies of the knee and its ability to demonstrate arthroscopically proved ACL tears and also compared MR findings with the findings of two commonly applied clinical tests of ACL instability, i.e., Lachman test and anterior drawer test. The sensitivity of MR imaging was 94% compared with 78% for AD test and 89% for the Lachman test. The specificity was 100% for all three.

Oberlander *et al*.,^2^ in their evaluation of the diagnostic accuracy of the clinical examination of the knee in 296 patients, found the sensitivity and specificity of the tests used to diagnose ACL injury to be 63 and 99%, respectively.

Benjaminse *et al*.,^6^ in a metaanalysis, reviewed 28 studies about the accuracy of clinical tests to diagnose ACL ruptures. The Lachman test showed a pooled sensitivity of 85% (95% confidence interval [CI] 83–87) and a pooled specificity of 94% (95% CI 92–95), but the range of sensitivity and specificity in these 28 studies was from 25 to 100%, which highlights the lack of uniformity. In the same study, the AD sign showed a pooled sensitivity and specificity in chronic ACL tear of 92% (95% CI 88–95) and 91% (95% CI 87–94), but the range was 2–100%. The pooled sensitivity and specificity for the pivot shift test in this study were 24% (95% CI 21–27) and 98% (95% CI 96–99).

Ostrowski,^7^ after a review of 17 studies about the accuracy of clinical tests for diagnosing ACL ruptures, concluded that a positive result for the pivot shift test is the best for diagnosing an ACL rupture, whereas a negative result to the Lachman test is the best for ruling out an ACL rupture. He also stated that the AD test is inconclusive for drawing strong conclusions either way.

Kocabey *et al*.^3^ compared the accuracy of clinical examination versus MRI in diagnosing meniscal and ACL pathology. The sensitivity of the McMurray test for diagnosing medial and lateral meniscus tear in the clinic was 87 and 75%, respectively. The specificity for the same two tests was 68 and 95%, respectively.

All the subjects in our study had non-acute ACL injuries where more than 3 weeks had elapsed from the time of injury. The sensitivity of the Lachman test in the OPD was 78.6% the specificity was 100% and the sensitivity and specificity of AD test in OPD were 89.3 and 100%. The sensitivity and specificity of the pivot shift test in OPD were 75 and 100%.

The above observations confirm the findings of Lee *et al*.^5^ and Katz *et al*.^8^ that the Lachman test, AD, and pivot shift tests are highly specific for ACL injury in a non-acute setting. The sensitivity of the AD test being more than that of the Lachman test can be explained on the basis of the fact that our series had a lesser number of medial meniscal injuries (50%) compared with other series (around 70%). The lower sensitivity of the pivot shift test compared with other tests could not be explained.

Examination under anesthesia revealed the sensitivity and specificity of the Lachman test to be 92.9 and 100%. The AD test's sensitivity and specificity were found to be 92.9 and 100% and pivot shift test's sensitivity and specificity were found to be 100 and 100%. These findings are better than those observed by Katz *et al*., who had sensitivities of 84.6, 53.8, and 84.6% for Lachman, AD, and pivot shift tests and a specificity of 95% for each.

Our study also showed that examination under anesthesia for Lachman test, AD sign, and pivot shift test is more sensitive than examination in the OPD, which confirms the findings of earlier studies.

The sensitivity of the McMurray test for medial and lateral meniscus in our study was 35.7 and 22.2%, respectively, and the specificity was 85.7 and 100%, respectively. Kocabey *et al*.,^3^ in their similar study, found a sensitivity and specificity of 87 and 68% for medial meniscus and 75 and 95% for lateral meniscus. Oberlander *et al*.^2^ reported that the McMurray test for medial meniscus had a sensitivity and specificity of 87 and 93%, respectively. The sensitivity and specificity of the McMurray test for lateral meniscus were 81 and 93%, respectively The lower sensitivity of the McMurray test in our study compared with the above two studies could be due to the fact that all the patients in our study had ACL injury.

## CONCLUSIONS

Lachman test, AD test, and pivot shift test are highly specific tests to diagnose ACL laxity in a non-acute setting. Pivot shift test under anesthesia is the most sensitive and specific test for diagnosing anterior cruciate ligament laxity in a non-acute setting. The McMurray test is not a sensitive test to diagnose meniscal injury in the presence of ACL injury.
